# Differences in Dyadic Coping Between Couples Facing Early-Stage Dementia Compared with Couples Facing a Visual Impairment and Healthy Couples

**DOI:** 10.1007/s10880-025-10124-x

**Published:** 2026-01-12

**Authors:** Peter Muijres, Josef Jenewein, Katharina Weitkamp, Fridtjof Nussbeck, Isabella Bertschi, Christina Breitenstein, Mona Neysari, Guy Bodenmann

**Affiliations:** 1 University of Zurich, Zurich, Switzerland; 2 Private Clinic Hohenegg, Meilen, Switzerland; 3 University of Konstanz, Konstanz, Germany

**Keywords:** Dyadic coping, Dementia, Visual impairment, Health impairment, Chronic illness, Couples

## Abstract

The concept of dyadic coping (DC) describes ways in which couples cope with stress together, including, for example, supportive DC (i.e., supporting a partner’s individual coping efforts) or delegated DC (i.e., taking over tasks from one’s partner). Previous research has primarily focused on DC in relation to chronic physical illnesses, foremost cancer. However, stress related to neurocognitive or physical conditions differs with regard to symptoms, course of progression, and associated psychological and social challenges. The current study aims to examine how DC in individuals with early-stage dementia differs from DC in individuals with a visual impairment or in a healthy condition, as well as how DC differs between their partners. Data from two studies were first matched with a sample of couples facing early-stage dementia (*n* = 37). Couples facing a visual impairment and healthy couples served as control groups. The Dyadic Coping Inventory was used to assess the DC exchanged. Analyses of variance and covariance as well as post hoc tests were used to test for differences in DC. Results show that partners of persons with early-stage dementia report receiving less supportive DC than partners in couples facing a visual impairment or healthy couples, as well as less delegated DC than partners in healthy couples. We did not find any differences on any DC domains in the persons with early-stage dementia compared to persons with visual impairment or persons in healthy couples. This study shows that DC of partners in couples facing early-stage dementia differs from partners in couples facing a visual impairment or healthy couples. Partners should avoid overprotective behaviors that could diminish the sense of self-efficacy of the person with early-stage dementia. Besides methodological suggestions, suggestions are made how tailored interventions, like facilitating participation and portioning tasks, may help improve DC, relationship functioning, and mental health.

## Introduction

The concept of dyadic coping (DC) explains how couples deal with stress together. The Systemic Transactional Model (STM) of stress and coping within couples presents the most nuanced understanding of dyadic coping (DC) (Bodenmann, 2005; Bodenmann et al., 2016). DC refers to the interactive process in which one partner expresses stress using verbal, paraverbal, or nonverbal cues, and the other partner responds accordingly with verbal and/or nonverbal behaviors (Bodenmann, 1995, 2005; Falconier & Kuhn, 2019). This process contributes to maintaining balance in the relationship by promoting mutual support and understanding during periods of stress, and it is a key predictor of relationship quality (Falconier et al., 2015).

DC consists of multiple components. Stress communication involves the degree to which the stressed partner expresses their distress and seeks help from their partner (e.g., “I ask my partner to do things for me when I have too much to do”). Supportive DC refers to the ways in which one partner actively helps the manage stress, such as offering empathy and emotional support. Delegated DC entails one partner alleviating the other’s stress by taking on certain responsibilities or tasks (e.g., “When my partner feels overwhelmed, I help by taking things off their plate”). Negative DC captures unhelpful or harmful responses, including hostility, ambivalence, or dismissiveness (e.g., “I don’t take my partner’s stress seriously”). Common DC involves both partners working together to address the stress of one partner, using either emotion-focused or problem-focused strategies (e.g., “We have an in-depth discussion about the issue and figure out what needs to be done”) (Bodenmann, 1997, 2005).

The interest in DC, both clinically and in research, has been steadily growing since its inception in the 1990s (Weitkamp & Bodenmann, 2022). Over the past three decades, research on DC has consistently demonstrated the positive impact of positive DC on physical health, well-being, relationship satisfaction, and couples’ adjustment to chronic illness, whereas negative DC is consistently linked to a negative impact (Landolt et al., 2023; Weitkamp et al., 2021). Reviews of studies on DC found that constructive communication of stress and positive DC (supportive, delegated, or common DC) alleviate couples’ stress and facilitate mental health in couples coping with cancer (Chen et al., 2021; Traa et al., 2015). Negative DC was associated with more relationship stress, lower relationship satisfaction, lower quality of life, and more depressive symptoms. Stress communication and positive DC were consistently found to increase relationship functioning, whereas negative DC lowered it (Bodenmann et al., 2011; Manceau et al., 2024). Expressing disapproval or critique or overprotecting the other partner was associated with more depression, and the exchange of supportive DC with less (Alves et al., 2018; Brandão et al., 2019; Hooley, 2007; Meuwly et al., 2012). DC aims to restore a state of homeostasis by addressing stress-provoking challenges. However, couples facing different health conditions may experience different types of stressors they need to deal with.

Specific illness-related challenges such as those presented by early-stage dementia or a visual impairment can significantly affect how couples engage in DC. These conditions introduce unique stressors and changes to the couple’s daily life, for example, by changing roles, communication, and perceptions of autonomy that require adaptation in the way partners support each other.

Dementia represents a particularly comprehensive challenge, comprising multiple life domains at once through a broad range of symptoms and side effects that affect couples and place high stress on caregivers (Campbell, 2009; Cooper et al., 2008; Häusler et al., 2016; Lee et al., 2019). Researchers identified stress, social changes, time perception, and uncertainty as the major challenges for couples facing neurodegenerative disorders, including early-stage dementia (Manceau et al., 2024). In general, stressors consist of concerns over dependency and the redivision of roles (Braun et al., 2009; Manceau et al., 2024; Muijres, 2021, 2023), while depressive mood, psychological distress, and additional health problems are also common (Azocar et al., 2021; Häusler et al., 2016). The available studies suggest that caring for individuals who developed early-stage dementia is associated with more negative outcomes for caregiving partners than caring for individuals with other disorders is (e.g., Cooper, 2008; Moise, 2004).

However, with regard to DC of couples facing dementia, mixed results were found. Higher DC is associated with lower levels of depression (Gellert et al., 2018; Landolt et al., 2023) and anxiety (Landolt et al., 2023) in individuals with early-stage dementia and their caregiving partners. Some studies found that couples benefit from fostering mutual understanding and positive DC (Bannon, 2021) as well as from empathy and mutual support exchanges in couples coping with early-stage Alzheimer’s disease (Huo et al., 2024). Higher emotion-focused common DC may reduce distress, whereas higher supportive DC from partners may also confront people with early-stage dementia with their decline, increasing their illness awareness and lowering their quality of life (Muijres et al., 2025). Similarly, other studies found that ‘positive’ DC was associated with more depressive symptoms in couples facing dementia (Connelly et al., 2020; Gellert et al., 2017). Especially in advanced stages, communication difficulties and diminished reflection hinder mutual responsiveness and common DC (O’Shaughnessy et al., 2010). The unpredictable progression of dementia further complicates adaptation (Henry et al., 2009) and presents a lasting challenge for both partners (Kristofferzon et al., 2018). The subjective caregiver burden—encompassing emotional, physical, social, and economic aspects—has been identified as a key determinant of clinically significant anxiety in family caregivers as well as of the quality of care provided (Del-Pino-Casado et al., 2021). Cognitive decline limits stress communication and problem-solving in people with early-stage dementia, which increases the caregiving burden for partners through delegated DC. Negative DC strategies, such as disengaged avoidance, may also help care-giving partners at times by keeping dyadic stress at bay (Colclough et al., 2023).

The conditions differ for couples facing a visual impairment. A visual impairment is a sensory dysfunction that does not directly affect physical health, cognitive, or executive functioning, but the limited capacity to navigate and respond to the visual aspects of the physical and social environment may be associated with social isolation and feelings of helplessness, depression, or loneliness (Nyman et al., 2012). These negative feelings may in turn compromise executive functioning (Nyman et al., 2012) and social life (Lehane et al., 2017). However, in visual impairment challenges arise mainly from reduced social participation, reliance on partner assistance, and difficulties in perceiving nonverbal cues (Bertschi et al., 2021a, 2021b). In comparison with couples facing neurodegenerative disorders, couples facing a visual impairment are coping with a sensory dysfunction and its consequences, but no neurodegenerative disorder that directly interferes with the neurocognitive foundation of executive and social functioning and is actively contributing to its accelerated decline.

How couples manage health-related needs and challenges influences relationship functioning, adaptation, levels of distress (Bodenmann et al., 2011; Falconier et al., 2015; Manceau et al., 2024), and both partners’ well-being (Del-Pino-Casado et al., 2021; Downward et al., 2022). However, most research on DC has focused on couples facing chronic physical illnesses, especially cancer (Weitkamp et al., 2021). Studies on DC in the context of mental health issues have only emerged since 2000, and the number of publications remains limited (Lyons et al., 2024; Weitkamp & Bodenmann, 2022). Consequently, little is known about how DC differ between couples confronting dementia-related challenges and those coping with other health conditions (Braun et al., 2009; Colclough et al., 2023). Since much of the existing literature focuses on chronic physical illnesses (Weitkamp et al., 2021), there is a clear need for more focused research on differences in DC in couples facing early-stage dementia and a visual impairment or healthy couples to better understand how DC and specific illness conditions are interrelated.

In this study, our aim was to explore the differences in DC of (index) persons with early-stage dementia compared to persons with a visual impairment or healthy persons (i.e., persons in a community sample of couples not facing notable chronic health conditions) as two control groups, as well as to compare the DC of partners in couples facing early-stage dementia to partners in couples facing visual impairment or in healthy couples. We considered persons with early-stage dementia to be in the most care demanding position associated with high caregiver stress, whereas healthy couples are unaffected by health problems and most likely to be in a symmetrical relationship. Related to a descending degree of dependency, persons with early-stage dementia were expected to receive the highest levels of supportive DC, delegated DC, negative DC, common DC, including problem- and emotion-focused common DC, followed by persons with a visual impairment and then by the index persons in healthy couples. Regarding the partners, a reverse order was expected with partners in couples facing early-stage dementia reporting to receive the least supportive, delegated, negative, and common (including emotion, as well as problem focused) DC first followed by partners in couples facing a visual impairment and then by partners in healthy couples.

## Methods

### Procedure

The data on DC of couples in the different health conditions of our study were collected in three different study projects. The data on couples facing early-stage dementia were collected between March 2019 and March 2021 as part of a study examining the feasibility, acceptance and benefits of Dignity Therapy in people with dementia and their relatives (the DTD study, Jenewein et al., 2021). Eligible participants for the trial were outpatients aged 18 years or older, diagnosed with early-stage dementia based on the Clinical Dementia Rating (CDR, Morris et al., 1993; Morris et al., 1997) with a score ranging from CDR ≥ 0.5 to CDR ≤ 1.5, proficient in the German language, and with a study partner (such as a romantic partner, relative, or close friend) willing to participate. Of the 54 dyads participating in total in the DTD -study, only 37 dyads were heterosexual romantic couples and included in the current study.

The data on couples facing vision loss stem from the SELODY study (Breitenstein et al., 2022), which aimed to better understand how vision affects couples in their relationship and what individual and dyadic factors benefit their DC. Inclusion criteria were couples with a) one partner having a severe visual impairment that had developed or significantly deteriorated during the current relationship, b) to be over 18 years of age, and c) speak one of the study languages (French, German, or Italian). In the SELODY study, DC was collected in 104 mixed gender couples with one partner living with severe vision loss. Study data were collected between May and December 2019. Data for the healthy sample were collected within the PASEZ study (Weitkamp et al., in prep.), a prospective longitudinal study over ten years. The PASEZ dataset included 368 healthy, mixed gender couples and aimed to assess the development of stress experiences, DC, and relationship satisfaction over time. Inclusion criteria were a) being in a romantic relationship with the current partner for at least one year, b) sufficient command of the German language, and c) being at least 18 years of age. Data were collected in three age cohorts. For the current analyses, data were used from the oldest cohort with 119 community-dwelling heterosexual couples aged 65 to 80 years. Compared with the SELODY and PASEZ studies, the sample size in the DTD study was the smallest (*n* = 37 couples) and the highest in age and set the standard that the other samples were matched.

### Matching Procedure

Couples from the SELODY sample were eligible if one person in the couple had a visual impairment only without other sensory impairments or chronic illnesses and one healthy partner (i.e., physically unaffected). Also, couples must have no more than 5% missing values.

**Fig. 1:**
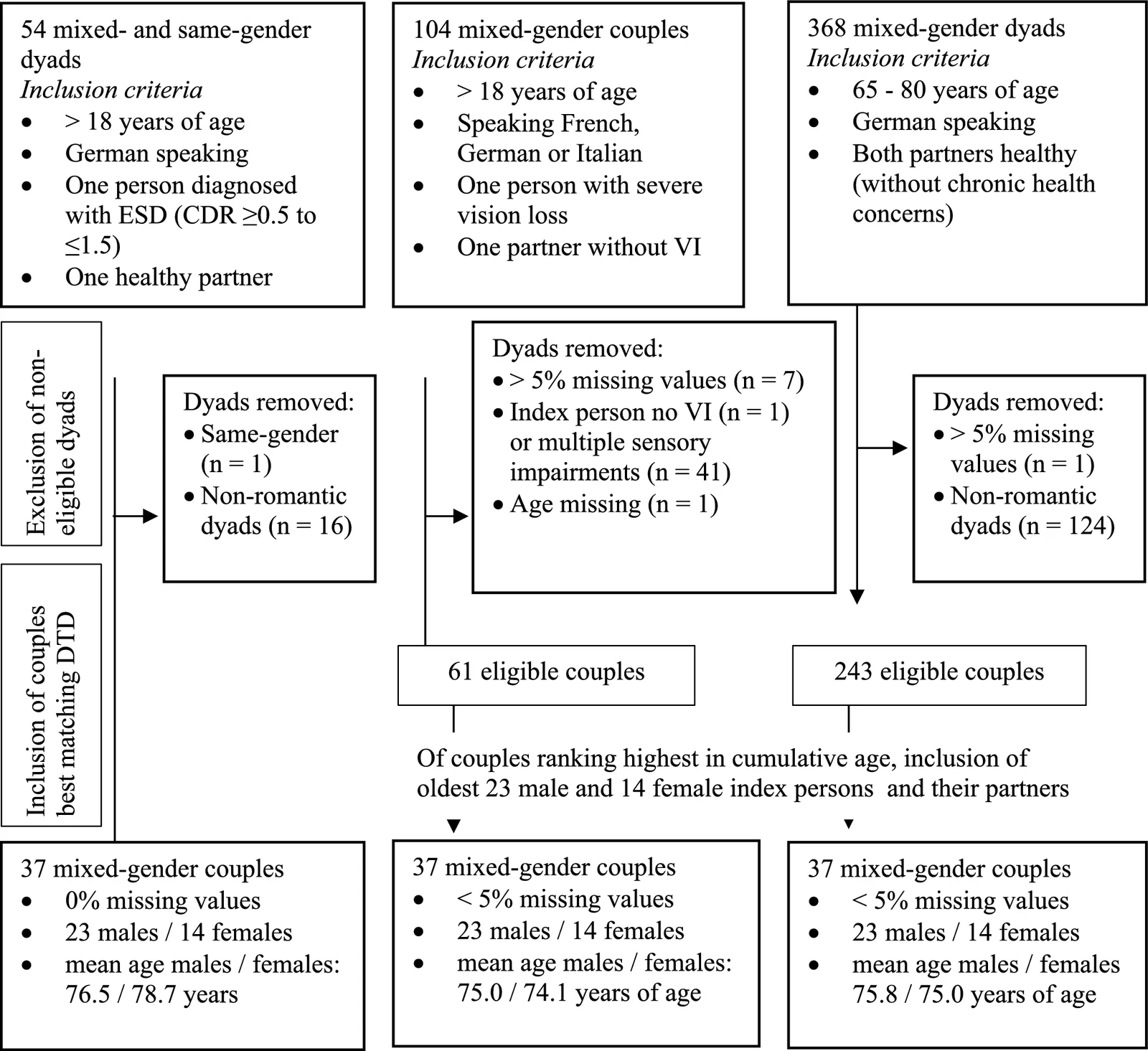
The parallel matching procedure couples in the original samples of study projects. *ESD* Early-stage dementia, *CDR* Clinical dementia rating (Morris et al.), *VI* Visual impairment, *DTD* the study project on dignity therapy with couples facing ESD, *SELODY* the study project on visual impairment in a dyadic context, *PASEZ* the study project on impact of stress on relationship development of couples and children

To replicate the gender distribution of the DTD sample (23 males, 14 females) and approximate the average age of couples facing early-stage dementia (*M* = 77.8 years, *SD* = 6.10), the oldest 23 males and 14 females with a visual impairment were assigned to the group ‘index persons with visual impairment,’ and their partners to the group ‘partners in couples facing a visual impairment.’ Lacking any distinguishing chronic physical or mental health condition, 23 male partners (62.2%) and 14 female partners (37.8%) from the 37 healthy couples with the highest average ages were randomly assigned to the group ‘index persons in healthy couples’ and their partners to the group ‘partners in healthy couples. The parallel matching procedure is depicted in Fig. 1.

### Participants

The samples in this study derived from unrelated research projects and the availability of demographic and questionnaire data, as well as the measures they were collected with, differed extensively. The mean age for men was *M* = 47.3 years (range: 19–80) and *M* = 49.3 years for women (range: 20–82). In most couples, partners had Swiss (84.0%), German (9.0%) ,or Italian (1.6%) nationality. Relationship duration averages around 21.0 years, and for the highest education level, 40.8% reported a vocational school apprenticeship, 31.5% higher education/university, 21.5% grammar school, and 5.4% lower education. Most participants (81.4%) had parents that never separated or divorced. Among the participants, 83.5% lives with their partner and 16.5% without, in the city (51.0%) or in the country (49.0%).

In the SELODY study, the average age for men was *M* = 58.9 years (range 31–93) and *M* = 58.4 years (range 28–89) for women. The average relationship duration is *M* = 30.5 years (*SD* = 17.96, range 2.2–69.9). The average income reported by participants is *M* = 57,000 Swiss Francs (*SD* = 45,152, range 2200–200,000). Of participants, 84% are married and 96% live in the same household with an average of *M* = 0.5 children per household (range 0–6). Limited common variables across the SELODY, PASEZ, and DTD studies constrained matching.

There were significant differences in mean ages of index persons between the samples *F*(2, 108) = 4.30, *p* = .016, *η*^*2*^ = .07. The values are listed in Fig. 1. Tukey’s post hoc test revealed that persons with early-stage dementia were significantly older than persons with a visual impairment (*p* = .017, *MD* = 3.84, CI 95% [0.06, 7.11]).

The mean age of the partners was *M* = 75.9 years, *SD* = 6.37. For partners in couples facing early-stage dementia: *M* = 78.8 years, *SD* = 5.86; partners in couples facing visual impairment: *M* = 74.1 years, *SD* = 7.46; and partners in healthy couples: *M* = 75.0 years. *SD* = 3.66). No significant differences were found between the mean ages of partners in different health condition groups.

Of 37 early-stage dementia couples, 36 were married (97.3%) for an average duration of *M* = 46.9 years (range 5.0–67.0, *SD* = 13.9). Of 37 couples facing a visual impairment, all were married (100%) for an average duration of *M* = 47.4 years (range 15.1–69.9, *SD* = 11.3). Of the 37 healthy couples, all were married (100.0%) as well with an average duration of *M* = 47.6 years (range 3.0–47.6, *SD* = 14.4).

### Measures

The Dyadic Coping Inventory (DCI; Bodenmann, 2008) was used to assess DC. The DCI is a 37-item self-report instrument that measures DC using a 5-point Likert scale ranging from 1 (*never/very seldom*) to 5 (*very often*). Each partner in a couple filled out the levels of DC they provided to and received from the other partner. The DCI was completed at baseline before participation in the Dignity Therapy intervention. The data collected in the study samples had only a select number of subscales in common.

In the SELODY study, data were collected in couples facing a visual impairment only using the DCI subscales *stress communication* (4 items), *received supportive DC* (5 items), *received delegated DC* (2 items), *received negative DC* (4 items), and *common DC* (5 items), including problem-focused common DC (3 items) and emotion-focused common DC (2 items) of all DCI subscales, limiting the comparison to these subscales. Means, standard deviations, and intercorrelations between subscales are listed in Table 1. With Cronbach’s alpha varying between *α* = .66 for negative DC and *α* = .90 for supportive DC received, the internal consistency of the DCI subscales varied between sufficient and excellent.

**Table 1:** Intercorrelations of the DCI subscales used for couples in all health conditions

			DCI subscale							
DCI subscale	*N*	*M*	SD	SC	SDCrec	DDCrec	NDCrec	CDC	CDCpf	CDCef
SC	222	3.3	.86	—	.39**	.41**	.12	.41**	.38**	.31**
SDCrec	222	3.5	.97		—	.62**	− .37**	.70**	.670**	.49**
DDCrec	221	3.5	1.05			—	− .16*	.43**	.39**	− .35**
NDCrec	222	1.91	.75				—	-.31**	-.29**	-.24**
CDC	222	3.39	.86					—	.88**	.84**
CDCpf	222	3.93	.89						—	.48**
CDCef	222	2.56	1.15							—

*DCI* dyadic coping inventory, *SC* stress communication, *SDCrec* received supportive, DC, *DDCrec* received delegated DC, *NDCrec* received negative DC, *CDC* common DC, *CDCpf* problem-focused CDC, *CDCef* emotion-focused CDC

### Statistical Analysis

We calculated a post hoc power analysis for the group index persons and for the group partners, each with a sample size of *n* = 111, for correlations assuming a medium effect. The DC data were normally distributed for index persons and partners in all samples, as assessed by the Shapiro–Wilk test (*α* = .05). For index persons, homogeneity of variance was asserted for all subscales (Levene’s test, *p* > .05). As the significant difference in age may violate the covariate balance, age of the index persons was included as a covariate in the analyses.

For partners, homogeneity of variance was asserted for subscales stress communication, negative DC, common DC, and problem-focused common DC (Levene’s test, *p* > .05), but missing in the subscales of supportive DC, delegated DC ,and emotion-focused common DC (Levene’s test, *p* < .05). Where homogeneity of variance was given, an ANOVA was used for the analysis, followed by Tukey post hoc tests to test for significant results. When homogeneity of variance was not asserted, the more robust Welch’s ANOVA was applied, and significant results examined using Games–Howell post hoc tests. Although all analyses are exploratory, we do not control for Type I error inflation due to the comparably small sample size and, hence, reduced statistical power. Statistical analyses were conducted with IBM SPSS Statistics 25.0.

## Results

### Index Persons

An analysis of covariance with age as covariate was conducted to test for significant differences between index persons. No significant differences were found for index persons on any of the DCI subscales. Results are shown in Table 2.

**Table 2:** DCI subscales compared between index persons in couples in different health condition groups

DCI subscales	Index persons in couples facing ESD		Index persons in couples facing VI		Index persons in healthy couples		*F*	*df1, df2*	*η*^*2*^
	*M*	*SD*	*M*	*SD*	*M*	*SD*			
SC	3.28	.95	3.24	.73	3.00	.81	.97	2, 108	.01
SDCrec	3.69	.78	3.66	.97	3.68	.66	.30	2, 108	.01
DDCrec	3.96	.88	3.93	.86	3.65	.89	.47	2, 108	.04
NDCrec	2.01	.72	1.87	.79	1.76	.70	.78	2, 108	.00
CDC	3.50	.82	3.40	.85	3.51	.79	.14	2, 108	.03
CDCpf	3.89	.83	4.01	.90	3.97	.70	.20	2, 108	.03
CDCef	2.92	1.22	3.37	.98	2.82	1.16	1.70	2, 108	.05

*DCI* dyadic coping inventory, *ESD* early-stage dementia, *VI* visual impairment, *M* mean, *SD* standard deviation, *F* F value*, df* degrees of freedom,* η*^*2*^ effect size, *SC* stress communication, *SDCrec* received supportive DC, *DDCrec* received delegated DC, *NDCrec* received negative DC, *CDC* common DC, *CDCpf* problem-focused CDC, *CDCef* emotion-focused CDC. Each subgroup contains *n* = 37 individuals. Calculated with ANCOVA

^*^p < .05; ** *p* < .01

### Partners

The received level of supportive DC differed significantly between the partners in couples in different health condition groups, Welch’s *F*(2, 70.74) = 5.90, *p* = .004, *η*^*2*^ = .11. The results are shown in Table 3. Confirming our expectations, Games–Howell post hoc analysis showed that received supportive DC scores of partners in couples facing early-stage dementia were significantly lower than those of partners in couples facing a visual impairment (mean difference = –0.64, 95% CI [–1.25, –0.03], Cohen’s* d* = -0.59) and lower than partners in healthy couples (mean difference = –0.86, 95% CI [–1.46, –0.26], Cohen’s* d* = –0.80). The statistical power of the comparison of supportive DC between partners in couples facing early-stage dementia and visual impairment was .54, which is relatively low. For the comparison between partners in couples facing early-stage dementia and healthy couples the power was .79.

**Table 3:** DCI subscales compared between partners in different health condition groups

DCI subscales	Partners in couples facing ESD		Partners in couples facing VI		Partners in healthy couples				
	*M*	*SD*	*M*	*SD*	*M*	*SD*	*F*	*df1, df2*	*η*^*2*^
SC^1^	2.79	.77	2.87	.93	3.02	.68	.79	2, 108	.01
SDCrec^2^	2.83	1.24	3.47	.92	3.69	.88	5.90*	2, 70.74	.11
DDCrec^2^	2.74	1.19	3.01	1.13	3.69	.71	10.53*	2, 66.77	.07
NDCrec^1^	1.97	.84	1.96	.72	1.89	.75	.12	2, 108	.00
CDC^1^	3.17	.99	3.22	.89	3.54	.80	1.78	2, 108	.03
CDCpf^1^	3.72	.99	3.84	1.02	4.12	.87	1.67	2, 108	.03
CDCef^2^	2.35	1.30	2.22	1.01	2.66	1.05	1.71	2, 70.74	.07

*DCI* dyadic coping inventory, *ESD* early-stage dementia, *VI* visual impairment,* M* mean, *SD* standard deviation, *F* F value, *df* degrees of freedom, *η*^*2*^ effect size, *SC* stress communication, *SDCrec* received supportive DC, *DDCrec* received delegated DC, *NDCrec* received negative DC, *CDC* common DC, *CDCpf* problem-focused CDC, *CDCef* emotion-focused CDC. Each subgroup contains *n* = 37 individuals

^*^p < .05

^1^Calculated with ANOVA

^2^Calculated with Welch’s test

The level of received delegated DC differed significantly for partners in different health condition groups, Welch’s *F*(2, 66.77) = 10.53, *p* < .001, *η*^*2*^ = .13. Games–Howell post hoc analyses showed that scores of delegated DC scores of partners in couples facing early-stage dementia were significantly lower, than those of partners in healthy couples (mean difference = −0.95, 95% CI [–1.49, –0.40]*,* Cohen’s *d* = −0.97), which also aligned with our expectations. The comparison of delegated dyadic coping between partners of persons with early-stage dementia and partners of persons with visual impairment had the highest power at .90, indicating a good ability to detect the observed effect.

## Discussion

Studies on DC in couples facing mental health challenges (Weitkamp & Bodenmann, 2022) and physical impairments (Bertschi et al., 2021a, 2021b) are sparse and no previous study has compared differences in DC of couples facing early-stage dementia with couples in other health conditions. The current study addressed this gap and explored differences in DC between the index persons in couples facing early-stage dementia and those in couples facing a visual impairment or in healthy couples, as well as differences in DC between their partners. We expected persons with early-stage dementia to receive and their partners to provide more frequent DC on all dimensions compared to persons with a visual impairment and healthy persons and their partners, respectively. Significant differences that confirmed our expectations were found in supportive and delegated DC. These differences showed that DC of partners varied according to the specific health condition affecting the couple. The findings provide preliminary new insights into the differences between DC of couples facing early-stage dementia in comparison with couples facing a visual impairment or healthy couples.

In couples facing early-stage dementia, partners that report receiving less delegated DC may perceive persons with early-stage dementia unable to take on chores. Besides problems with executive functioning or depressive symptoms, the extent of opportunities for persons with early-stage dementia to take on tasks may be limited by partners’ overprotective behavior, which has been described as dysfunctional in previous studies (see Coyne & Fiske, 1992). Although diminished neurocognitive functioning, associated with a reduced reflective awareness, could partly explain why partners in couples facing early-stage dementia report receiving relatively few DC, that assumption may lead caregiving partners to take over too many tasks and responsibilities too early. This tendency bears the risk of assigning persons with early-stage dementia to a passive patient role involving health risks, such as feelings of worthlessness, anxiety and depression (Asmer et al., 2018; Azocar et al., 2021) and a faster progression of dementia decline (Cheng, 2016).

When persons with dementia start demonstrating noticeable difficulties with reflection, judgment and executive functions, and their dependency increases, caregiving partners may start questioning the value of advice or assistance they have to offer. Dependency contributes to a loss of dignity in care receivers (Chochinov, 2005) and receiving help one-sidedly may induce guilt (Brown et al., 2003). Being cared for can harm feelings of self-esteem, self-efficacy, and dignity in persons with dementia (Leuchtmann & Bodenmann, 2017; Muijres, 2021, 2023). Unfortunately, partners in couples facing early-stage dementia may not always sufficiently see why, know how, or feel motivated enough to involve persons with early-stage dementia in problem-solving and things to do, which supports their sense of feeling appreciated. The psychological importance of opportunities to participate may go forgotten as partners struggle to come to terms with the emotional and practical challenges or presumed social expectancies of a radically changing situation.

Higher depression rates may also explain why partners in couples facing early-stage dementia report less DC received. Depressive symptoms are linked to reduced DC exchange between partners (Gellert et al., 2017). Depressive symptoms might demotivate both members in couples facing early-stage dementia to support the other partner in their problem-solving efforts.

Depressive symptoms and discontent among care receivers have been suggested as an indication of well-intended, yet ill-administered support (Braun et al., 2009). Another study with the same sample of couples facing early-stage dementia as was used in this study has shown discrepancies in the levels of supportive DC and delegated DC that persons with early-stage dementia claimed having provided and their partners acknowledged having received (Muijres et al., 2023). Partners taking care of persons with early-stage dementia may not acknowledge receiving DC from them when they do not experience their DC as helpful, whereas the afflicted persons may perceive and declare their DC efforts as substantial contributions.

An informed renegotiation of roles, wherein the remaining abilities of persons with early-stage dementia and the limits of their partners are accounted for, is important in order to economize caregivers’ resources. It is also crucial to invite persons with early-stage dementia to participate in shared responsibilities, since imbalances in support exchange are linked to dyadic distress and potential relationship conflicts (Acitelli & Badr, 2005; Braun et al., 2009) as proposed in the concept of “we-disease” (Leuchtmann & Bodenmann, 2017). Partners in couples facing early-stage dementia, who overextend themselves in their DC efforts, expose themselves to a risk of physical and mental exhaustion, while jeopardizing the quality of care and well-being of both partners (Cooper et al., 2008; Del-Pino-Casado et al., 2021).

## Limitations

This study has been the first to explore differences in DC between couples facing early-stage dementia compared to couples facing a visual impairment and healthy couples, but there are several limitations that need to be noted. First, findings are based on questionnaires, and no observational data were gathered, thus biases related to self-reports are possible. Although only persons in an early stage of dementia participated, dementia-related changes may have compromised their reports. Although the early stage of dementia did not appear to hinder persons in their comprehension of items, this option cannot be ruled out and could possibly explain why no significant differences between index persons were found, besides the methodological issues inherent to this study.

Using samples from three unrelated study projects severely compromised the options for matching. Propensity score matching as the preferred matching procedure to reduce selectivity bias failed, due to the lack of comparable variables. Variables were either not available in all three studies, measured with different instruments or data were stored in mixed formats, including text and numerical formats. As an attempt of unification would introduce a new bias, the available variables were limited to gender, age, absence of missing values, and availability of a healthy partner in all couples, which made parallel matching the method of choice.

The parallel matching also reduced the available sample size for the statistical analyses. This in turn forced us to choose between the correction of Type I error inflation or statistical power. Since this study is the first to investigate DC in three different health condition groups, we chose not to compromise statistical power and call for replications of our results based upon larger samples instead.

Whereas the current study examined differences in DC between groups in different health condition groups cross-sectionally, understanding how couples adapt their DC to changing conditions as dementia progresses, would have added useful insights. For example, both a reduced awareness as well as an attempt to retain a sense of dignity could explain why persons with early-stage dementia may deny that executive functions have deteriorated. The samples were limited in the number of variables they had in common, dramatically reducing the possibilities to account for psychological, social, economic, and cultural factors that may influence the role of DC within different health conditions. To what extent symptomatic changes interact with changes in DC in the context of couples facing early-stage dementia deserves further examination in future studies.

Another limitation is the failure to consider the role depression might play. Associated with an awareness of daily difficulties and cognitive decline, as well as an ‘anticipatory grief’ about losses to come, individuals with early Alzheimer’s Disease often develop depressive symptoms, which enhances their psychological distress and burden (Azocar et al., 2021). Depressive symptoms are found in as much as 20% in persons with dementia (Asmer et al., 2018; Savva et al., 2009). Prevalence of depression in visually impaired and healthy people is lower, with reported rates of 13.5% and 4.6%, respectively (Evans et al., 2007). As depressive symptoms may reduce cognitive awareness and coping abilities, differences in DC and quality of life might also be partly attributable to differences in depression.

Although it would have been interesting to understand how DC patterns diverge during the adjustment process to different health conditions, available data do not allow for a longitudinal comparison between our study samples. In addition, the increasingly questionable validity of self-report data in advanced stages of dementia poses an impediment to a longitudinal examination.

### Clinical Implications and Future Research

This exploratory study has been the first to observe that DC of partners in couples facing early-stage dementia differs from DC of partners in couples facing a visual impairment or healthy couples. Understanding the variations in supportive and delegated DC may help healthcare professionals, policy makers, and researchers prevent dysfunctional health-specific adaptations, like protective buffering or not addressing stress as a couple, and thus improve mental health and relationship functioning in couples in different health conditions.

Future research may wish to iron out methodological limitations inherent to this exploratory study by ensuring the collection of identical demographic and questionnaire data and using identical measures across all samples to facilitate a cross-sectional comparison. In addition to the availability of comparable variables, a larger sample of couples facing early-stage dementia would enable propensity score matching and thereby enhance the rigor of the matching process.

Larger samples would also aid future studies with higher statistical power. While some comparisons had adequate statistical power, others were underpowered. This underscores the need for caution in interpreting the results, particularly for the comparisons of supportive DC between partners in couples facing early-stage dementia and visual impairment. Replicating and possibly expanding our findings with larger sample sizes in future studies would increase confidence in the observed effects and potentially uncover smaller effects that may have been missed due to limited power.

Furthermore, the collection of qualitative data may add an additional understanding and help interpret the differential associations of DC with mental health in different health condition groups. As dementia progresses, the extent of opportunities for persons with early-stage dementia to provide practical support diminish. When persons with early-stage dementia accept and understand how to cope with a progressing neurodegenerative disorder, feelings of frustration may lead to an urge to overcompensate or may result in depressive symptoms (Azocar et al., 2021). According to the systemic transactional model, the individual distress of a person with early-stage dementia is likely to increase the distress in both partners, as well as the stress of the dyad as a unit. The findings of this study may tailor approaches like the Couple Communication Enhancement Training (Bodenmann & Shantinath, 2004) to the specific needs of early-stage dementia couples, for example by helping partners to recognize and harness the DC potential that the index person may be able to contribute. Should partners take over too many tasks too early, they may increase the DC they receive and decrease the care burden by learning how to facilitate persons with early-stage dementia to contribute. A breakdown of tasks into smaller subtasks, lower performance-oriented expectations and a relativizing response, when mistakes are made, could help relieve the need to uphold a sense of dignity and self-efficacy when dementia start compromising a person’s ability to help their partner out. Partners may receive more supportive DC, when learning to provide constructive feedback and showing appreciation themselves, thereby bolstering feelings of self-efficacy and harness helpful DC in persons with early-stage dementia.

## Data Availability

The data presented in this study are available on request from the corresponding author. The data are not publicly available due to ethical guidelines.
